# Effect of Prepreg Composition on the Structure and Shear Strength of PEI/CF Laminates Fabricated by Ultrasonic Additive Manufacturing

**DOI:** 10.3390/polym17111468

**Published:** 2025-05-25

**Authors:** Defang Tian, Vladislav O. Alexenko, Dmitry Yu. Stepanov, Dmitry G. Buslovich, Alexey A. Zelenkov, Sergey V. Panin

**Affiliations:** 1 Department of Materials Science, Engineering School of Advanced Manufacturing Technologies, National Research Tomsk Polytechnic University, 634050 Tomsk, Russia; defan1@tpu.ru; 2 Laboratory of Mechanics of Polymer Composite Materials, Institute of Strength Physics and Materials Science, Siberian Branch of Russian Academy of Sciences, 634055 Tomsk, Russia; vl.aleksenko@mail.ru (V.O.A.); sdu@ispms.ru (D.Y.S.); alexeyzelenkov@yandex.ru (A.A.Z.); 3 Laboratory of Nanobioengineering, Institute of Strength Physics and Materials Science, Siberian Branch of Russian Academy of Sciences, 634055 Tomsk, Russia; buslovich@ispms.ru

**Keywords:** polyetherimide, laminate, thermoforming, ultrasonic additive manufacturing, Taguchi method, artificial neural network modeling

## Abstract

In this study, laminates based on polyetherimide (PEI) with contents of carbon fibers (CFs) from 55 to 70 wt.% were fabricated by thermoforming (TF) and ultrasonic additive manufacturing (UAM) methods. The UAM laminates with CF contents above 55 wt.% possessed shear strengths lower by 40% in comparison with those of the TF ones, due to insufficient amounts of the binder in the prepregs to form reliable interlaminar joints. For enhancing the shear strength of the laminates with a CF content of 70 wt.%. up to the levels of the TF ones, extra resin layers with thicknesses of 50, 100, and 150 μm were deposited. By ranking the UAM parameters using the Taguchi method, it was possible to increase the shear strengths by 30% as compared to those of the trial laminates. Further improvements were achieved by artificial neural network (ANN) modeling. As a result, the use of the 50 µm thick extra resin layer made it possible to increase the shear strengths up to 50% relative to those of the trial laminates at a CF content of 70 wt.%. This improvement was achieved via minimizing the number of defects at the interlaminar interfaces. The dependences of both mechanical and structural characteristics of the laminates on the UAM parameters were essentially nonlinear. For their analysis and optimization of the UAM parameters, the direct propagation neural networks with the minimal architecture were utilized. Under the ultra-small sample conditions, the use of a priori knowledge enabled us to predict the results rather accurately.

## 1. Introduction

The global market for carbon fiber reinforced polymers (CFRPs) has shown significant growth over the past two decades, exceeding US$40 billion in 2025 [1]. CFRPs are widely used in numerous industries, such as wind energy, aerospace, automotive, construction, etc. [2]. According to their matrix types, CFRPs are classified as thermosetting and thermoplastic ones. Most products and structural elements are manufactured using CFRPs with thermosetting matrices/binders. Despite their higher cost, ones based on thermoplastic binders possess a number of advantages: unlimited shelf life, great processability, recycling capabilities, maintenance of strength characteristics at elevated temperatures, and high fracture toughness. At the same time, CFRPs based on thermosetting binders/matrices are not recyclable, and their disposal is an expensive and environmentally unfriendly technology [3].

A common industrial method for manufacturing products from CFRPs involves the fabrication of prepregs (i.e., bundles of carbon fibers (CFs) or CF-fabrics, pre-impregnated with a polymer binder). In the production of such laminates, thermoforming (TF), autoclave molding and automated laying techniques are implemented. In the latter case, a distinction is made between automated tape laying (ATL), in which a wide prepreg tape is laid out on a form (mandrel), and automated fiber placement (AFP), in which several narrower tapes are laid simultaneously. However, new techniques for producing CF-reinforced laminates are also under development, including their additive manufacturing [4,5,6,7].

Some disadvantages of the TF method include long durations of heating, holding, and cooling, as well as both cost and dimensions of molds, which limit the manufactured product sizes.

The autoclave molding technique also possesses a number of drawbacks. In particular, it requires great financial costs for both the fabrication and operation of equipment, as well as high energy consumption; the manufactured product sizes are limited by the autoclave chamber dimensions; most of the energy is spent on heating the equipment and tooling; it is impossible to promptly control the heating and forming of parts; and the heterogeneity of the laminate structures because of temperature gradients during the binder polymerization and subsequent cooling [8].

The ATL and AFP methods imply the use of various types of heaters: hot gas torches, infrared and pulsed light heaters, as well as lasers [9]. A significant issue, preventing their wide industrial applications, is the difficulty of obtaining defect-free laminate structures. The reason is the complete melting of polymer binders for joining layers and compacting CFs in the automatic laying process. This challenge results in polymer overheating and destruction, the formation of pores, waviness, low interphase and interlaminar adhesion, etc. [10,11,12].

One of the promising ways to solve the above-mentioned issues in the production of laminates is the implementation of ultrasonic additive manufacturing (UAM), based on the ultrasonic welding (USW) principles. USW is one of the most common methods of joining thermoplastics. In this way, high-frequency low-amplitude mechanical vibrations are applied for the frictional heating and local melting of contacting surfaces with the subsequent formation of a permanent joint [13]. USW allows joining both amorphous and semi-crystalline thermoplastic polymers without additional external heating [14]. Its additional advantages are both high efficiency (since its durations are in the range from fractions to several seconds) and the ease of the process automation in serial manufacturing.

In USW, the formation of a joint is based on the insertion of an energy director (ED) between the surfaces of welded parts, which is melted due to frictional (viscoelastic) heating and is partially squeezed out of the fusion zone, wetting the contacting surfaces [15,16,17]. In this scientific area, research efforts have been focused on several tasks: the optimization of parameters, the monitoring of which made it possible to control the formation of ultrasonic welded joints, minimizing damage to their components; and the selection of materials for manufacturing EDs, including controlled porosity, surface microtexturing (perforation), variations in shapes and thicknesses, etc. [13,18,19].

Recently, it was proposed to form an extra resin layer on one of the surfaces of the components to be joined [20,21]. It promotes more reliable adhesive bonding of adjacent layers, improves the quality and homogeneity of the interface, and acts as a thermal barrier to prevent prepreg overheating and damaging. This approach enables the formation of welded joints of both similar and dissimilar laminates based on polyetherimide (PEI) [22], as well as polyetheretherketone (PEEK) and epoxy [23,24], as examples.

The aim of this study was to optimize both UAM parameters and polymer content in the prepregs that enabled to form PEI-based laminates with homogeneous structure and improved mechanical properties. The null hypothesis was the possibility of forming homogeneous structure at the interlaminar interfaces, enhancing their shear strength through the use of prepregs with an extra resin layer, playing a role similar to EDs in USW.

## 2. Materials and Methods

### 2.1. Materials

The commercially available ‘Solver PEI ROOH’ powder (T&T Industry Group Ltd., Shenzhen, China) was used for the fabrication of prepregs, while the ‘ACM C285S’ bidirectional CF-fabric with a surface density of 285 g/cm^3^ (UMATEX, Moscow, Russia) was utilized for their reinforcement.

### 2.2. Prepreg Fabrication

**Figure 1:**
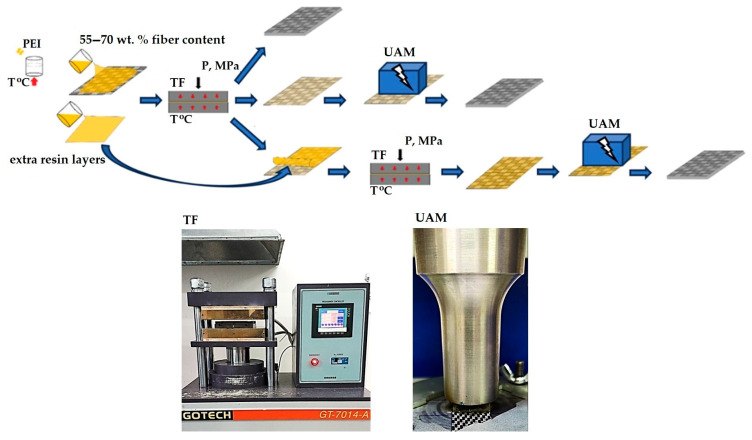
The schematic diagram of the prepreg fabrication process.

During the fabrication of prepregs, the CF contents were varied according to the data presented in Table 1, which also includes their thicknesses. A schematic diagram of the prepreg fabrication process is shown in Figure 1. Initially, rectangular shaped fragments of the CF-fabric were mechanically cut and placed in a solution of the PEI powder in N-methylpyrralidon (C_5_H_9_NO) for impregnation. Then, the solvent was evaporated in a drying oven (Memmert UN 30, Memmert GmbH, Schwabach, Germany) at a temperature of 170 °C for 6 h. Such ‘sungle-layer’ blanks were inserted in a press mold and subjected to hot pressing with a thermal press, a ‘GT-7014-A 122’ (GOTECH Testing Machines Inc., Taiwan, China), at a temperature of 360 °C and a pressure of 10 MPa.

**Figure 2:**
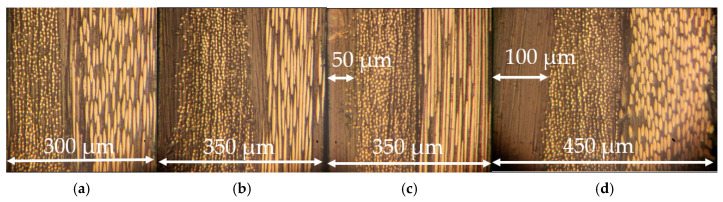
The cross-section photographs of the original prepregs with CF contents of 70 wt.% (**a**) and 65 wt.% (**b**), as well as ones with extra resin layers of 50 µm (**c**) and 100 µm (**d**).

To deposit extra resin layers, PEI was dissolved in N-methylpyrralidon according to the optimal proportion. Then, the polymer solution was poured into a glass cup and the solvent was evaporated, forming films with thicknesses of 50 ± 10, 100 ± 10, and 150 ± 10 μm. After that, the PEI films were hot pressed onto the surfaces of the prepregs with a CF content of 70 wt.% at a temperature of 360 °C and a pressure of 1.5 MPa. Figure 2 shows photographs of the characteristic cross section of the prepregs.

Relative thickness changes (*Rt*) were calculated using the results of measuring the samples’ thicknesses as follows:

(1) $$Rt=\frac{∆d}{d},$$

where d was the prepreg thickness, and ∆d was the prepreg thickness change.

### 2.3. Laminate Thermoforming

**Figure 3:**
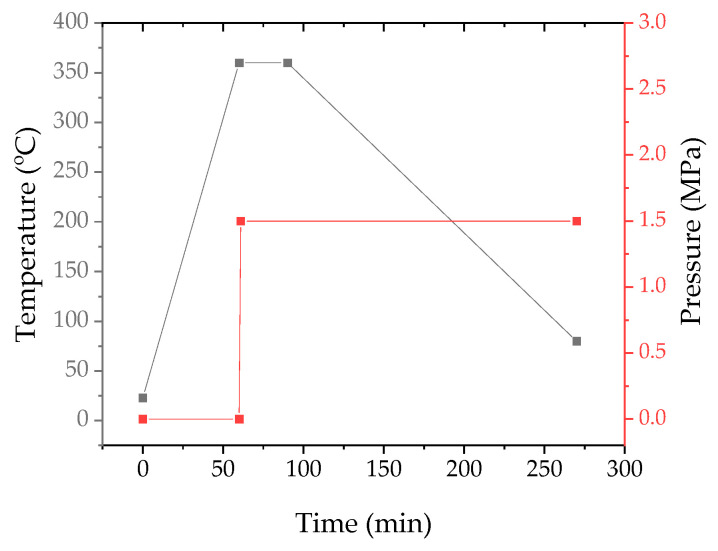
The time diagram on variations of temperature and pressure in the laminate thermoforming process.

For the fabrication of laminates from six laid prepregs, a TF procedure was implemented, which provided for changes in both temperature and pressure according to the scheme shown in Figure 3.

### 2.4. UAM Procedures

**Figure 4:**
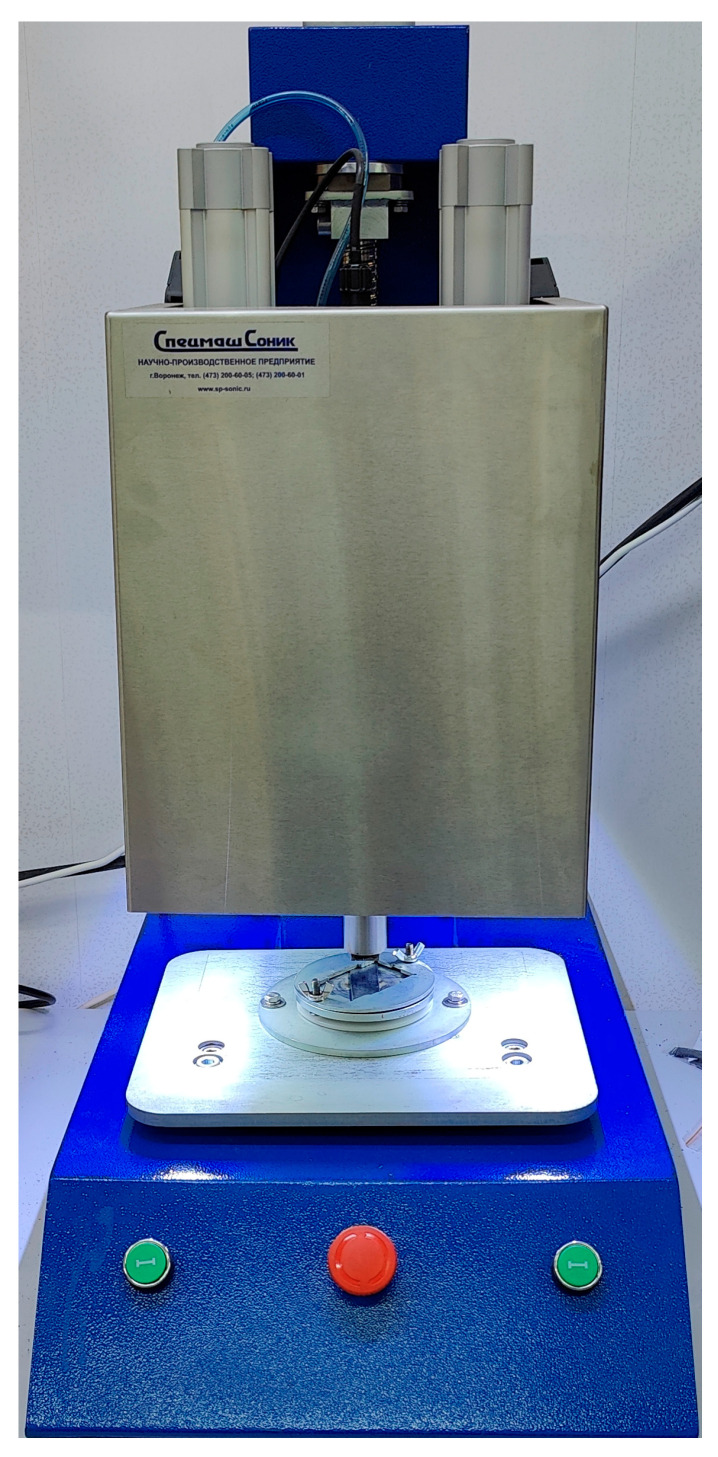
USW machine ‘UZPS-7’.

In the UAM procedures, a ‘UZPS-7’ USW machine (SpetsmashSonic LLC, Voronezh, Russia) was used at an oscillation amplitude of 10 µm and a frequency of 20 kHz. The sonotrode sizes and, accordingly, the fusion zone dimensions were 20 × 20 mm^2^ (Figure 4).

The Taguchi method [25] was used to plan the experiment. The input data were ranged, combining technological factors and their levels in the L9 format (Table 2).

### 2.5. Mechanical Tests

**Figure 5:**
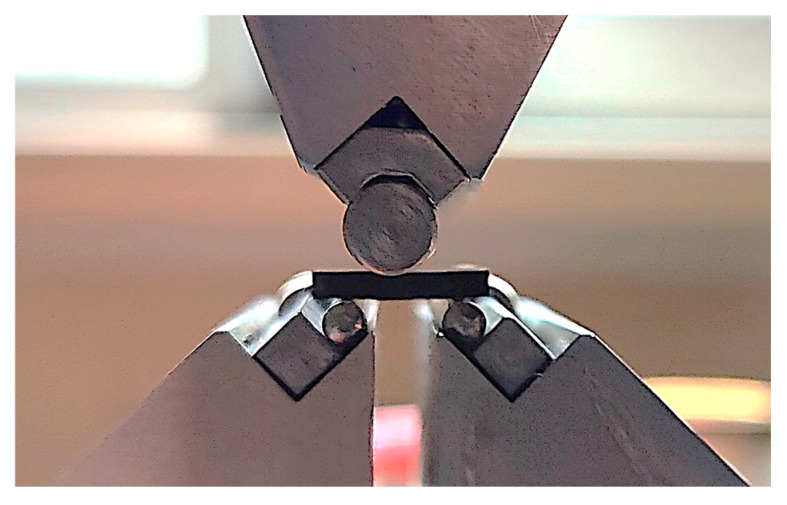
The installation for the three-point bending test.

Apparent ultimate shear strengths were estimated using the results of three-point bending tests of short beams (12 × 4 × 2 mm^3^), which were carried out according to ASTM D2344 with an ‘Instron 5582’ electromechanical machine (Instron, Norwood, MA, USA). The distance between supports was 8 mm, while the movable grip speed was 1 mm/min. The installation for clamping of the specimen is depicted in Figure 5.

### 2.6. Structure Characterization

The structures of the laminates were analyzed over their cross-sections with a ‘Neophot 2’ optical microscope (OM; Carl Zeiss, Jena, Germany) equipped with a ‘Canon 700D’ digital single-lens reflex camera (Japan) and a ’LEO EVO 50’ scanning electron microscope (SEM; Carl Zeiss, Germany) at an accelerating voltage of 20 kV. In this way, copper films were pre-sprayed onto the laminate surfaces to make them conductive.

## 3. Results and Discussion

### 3.1. Varying Binder Contents in the Prepregs

**Figure 6:**
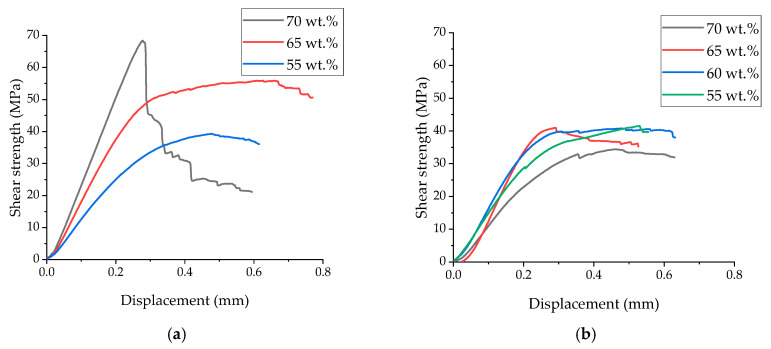
The displacement–shear strength diagrams for the TF (**a**) and UAM (**b**) laminates.

**Figure 7:**
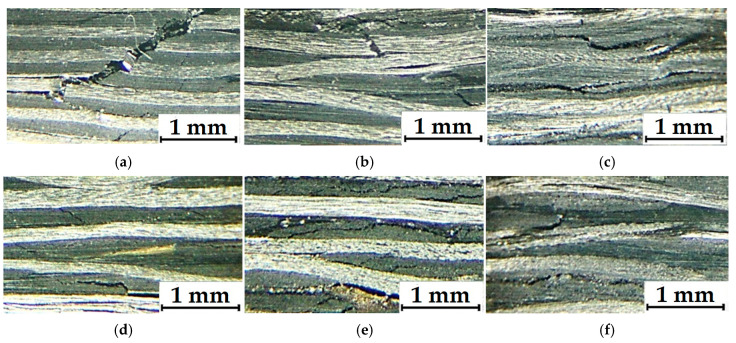
The optical photographs of the cross-sections the TF (**a**–**c**) and UAM (**d**–**f**) laminates at the CF contents of 70 wt.% (**a**,**d**), 65 wt.% (**b**,**e**), and 55 wt.% (**c**,**f**) after the three-point bending tests.

Figure 6 shows the “displacement–shear strength” diagrams recorded in the three-point bending tests, while photographs of the cross-sections of the TF and UAM laminates are presented in Figure 7. According to Figure 6a, the failure mode of the TF laminates changed with increasing their CF contents. At the lower levels of 55 and 65 wt.%, their loading was accompanied by plastic strains. In these cases, the maximum stresses were reached at displacements of 0.46 and 0.66 mm for the CF contents of 55 and 65 wt.%, respectively. With the loading, stresses gradually decreased, but cracks initiated and propagated predominantly along the interlaminar interfaces (Figure 6b,c). At a high CF content of 70 wt.%, the fracture was predominantly brittle taking place at a stress of 70 MPa. As shown in Figure 7a, the main crack propagated through several prepregs.

For the UAM laminates (Figure 6b), loading was also accompanied by plastic strains regardless of the CF content. In these cases, the maximum stresses barely exceeded 40 MPa, which was slightly lower than those for the TF ones with a CF weight fraction of 55%. According to Figure 7d–f, cracks initiated and propagated predominantly along the interlaminar interfaces.

**Figure 8:**
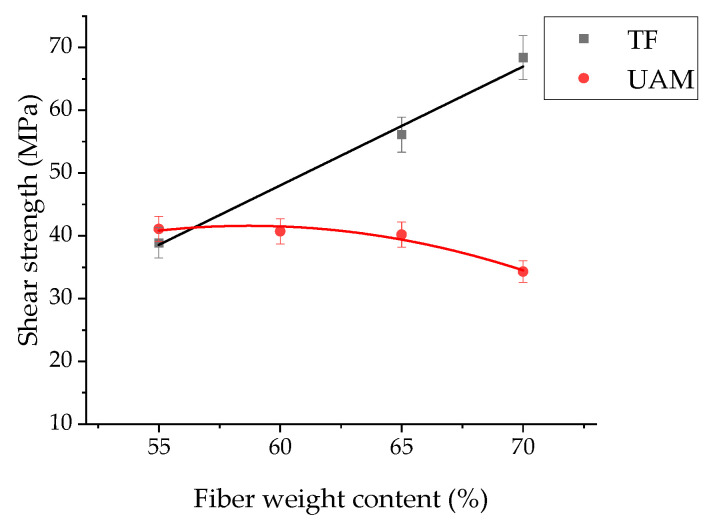
The shear strength versus CF content dependences for the TF and UAM laminates.

Figure 8 shows the results of the evaluation of the apparent ultimate shear strengths of the studied laminates. At the CF content of 55 wt.%, their values were similar for the TF and UAM laminates, but they increased linearly with enhancing the CF contents in the TF ones. The observed trends of the curves were in good agreement with previously published data [26]. At the same time, such curves were not linear in all cases, since they depended, among other things, on the amount of both matrix/binder materials and reinforcing CFs. For the UAM laminates, the apparent ultimate shear strengths just slightly lowered as the CF content increased. According to the authors, the reason was the insufficient amount of the binder in the prepregs compared to the level required for reliable bonding of the layers.

**Figure 9:**
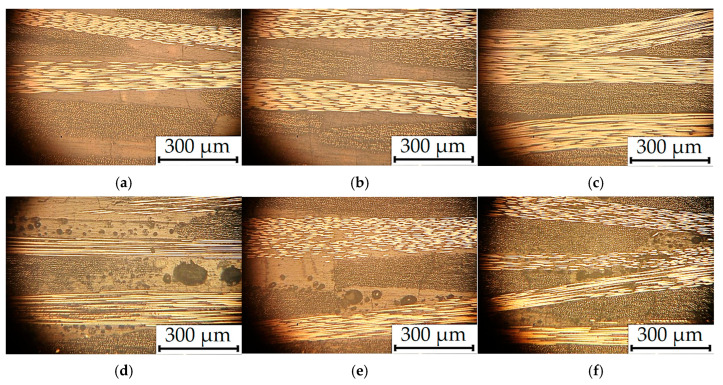
The optical photographs of the cross-sections of the TF (**a**–**c**) and UAM (**d**–**f**) laminates at the CF contents of 60 wt.% (**a**,**d**), 65 wt.% (**b**,**e**), and 70 wt.% (**c**,**f**).

Figure 9 presents photographs of the cross-sections of the TF and UAM laminates with different CF contents. In the TF ones, homogeneous defect-free structures were observed (Figure 9a–c), while pores were found at the interfaces between layers in the UAM laminates and in the CF-fabric interlacing zones (Figure 9d–f). At low binder contents, a probable cause of such defects was the impossibility of complete filling of voids between contacting layers with polymers during the UAM process. In addition, pores could be formed due to intense (turbulent) melted binder flows, including through CF-fabric layers, in the opposite cases.

**Figure 10:**
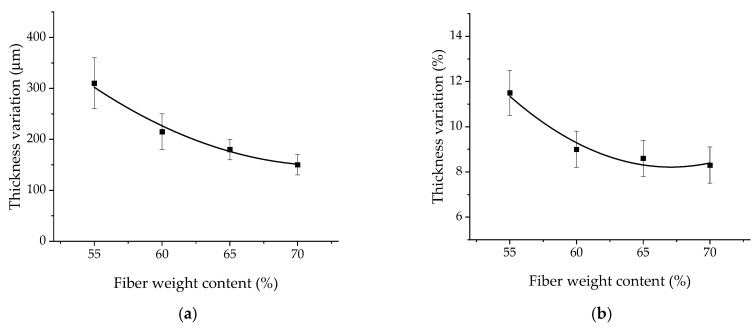
The thickness change versus CF content dependences for the UAM laminates: total (**a**) and in percent (**b**).

Figure 10 shows graphs on the thickness changes of the UAM laminates upon their manufacturing. Enhancing the CF content was accompanied by a nonlinear decrease in the relative thickness changes, which were almost identical in the range of 60–70 wt.%. This fact indicated that CFs had prevented the leakage of the molten binder from the stack of the laid prepregs at these CF contents.

In the UAM process, the thickness changes were caused by squeezing out the molten binder from the fusion zones. Since the component ratios were changed relative to the initial levels, this phenomenon exerted a negative effect. It could be eliminated by forming the extra resin layers on the prepreg surfaces that would be melted, joining the adjacent surfaces and preserving the initial component contents in the prepregs.

To conduct the research within such a statement, the prepreg with a high CF content of 70 wt.% was selected. Despite it not contributing to the greatest characteristics of the UAM laminates, it was promising for using with the extra resin layer. It was inappropriate to insert the prepregs with the lower CF contents, since their large amounts of the binder could not allow achieving high mechanical properties.

### 3.2. Varying Extra Resin Layer Thicknesses

The extra resin layers with the thicknesses of 50, 100, and 150 µm were deposited on one side of the prepregs. If they have been completely pressed out (spread), the thickness changes are to make 300 µm for samples No. 1–3, 600 µm for No. 4–6, and 900 µm for No. 7–9.

**Figure 11:**
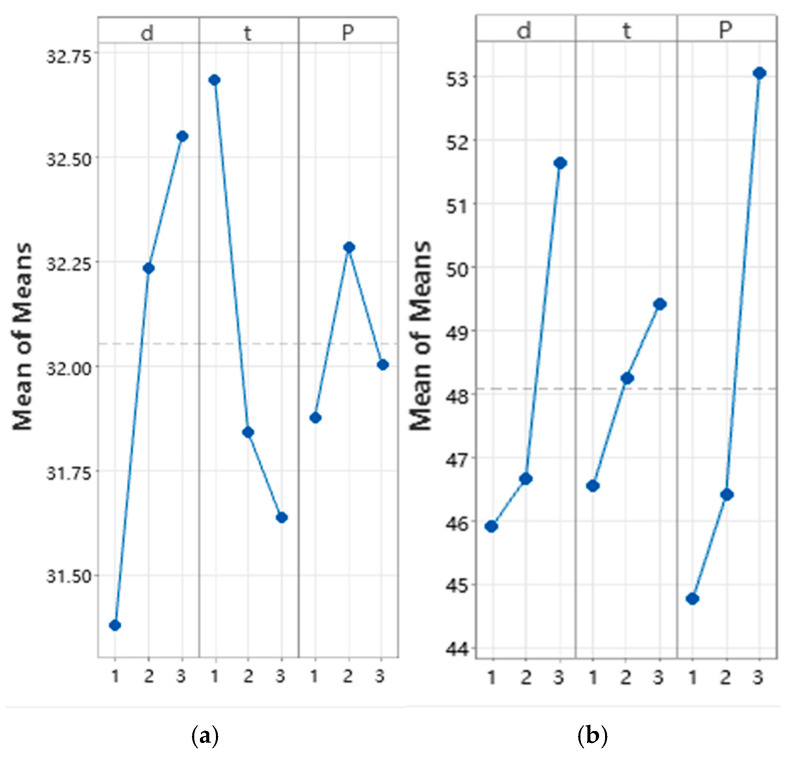
The S/N ratios for different levels of the UAM parameters: (**a**) shear strength; and (**b**) thickness change.

The experiment was planned and data were processed according to the Taguchi method (Table 2). The signal-to-noise ratio (S/N) was interpreted in terms of ‘the higher, the better’. Table 3 presents the results of the evaluation of both shear strengths and thickness changes after the UAM procedures. Each value was obtained by averaging over four experimental points. A preliminary analysis of these results showed that the ranges of variations of the input parameters did not result in a multiple change in the properties of the laminates. According to their ranking, summarized in Table 4, the factor with the maximum influence had the minimum number. Figure 11 shows changes in the output parameters of the UAM process relative to the input data variations, which enabled us to assess the nature of their mutual influence and select the rational combinations.

It was found that the greatest contribution to the increase in the ‘shear strength’ output parameter was made by both the ‘extra resin layer thickness’ and ‘UAM duration’ input ones. The ‘thickness change’ output parameter was more influenced by the ‘clamping pressure’ and ‘extra resin layer thickness’ input ones. However, the analysis of these data by the Taguchi method did not allow either to determine the exact (not used in the experiments) values of the input parameters or to give an unambiguous interpretation of the reasons for their influence. Therefore, it was necessary to apply an alternative approach that enabled us to more objectively optimize the UAM parameters, by examining the structural characteristics as an instance.

**Figure 12:**
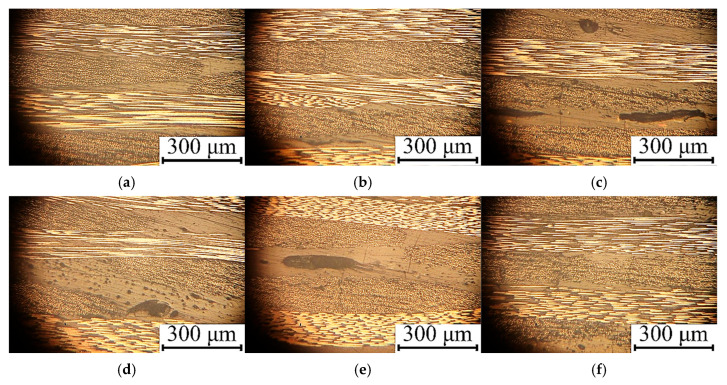
The optical photographs of the cross-sections of the UAM laminates fabricated using modes 1 (**a**); 5 (**b**); 4 (**c**); 7 (**d**); 9 (**e**); and 3 (**f**).

Figure 12 presents photographs of the cross-sections of the UAM laminates fabricated using modes 1, 3, 4, 5, 7, and 9. For modes 1 and 5, defect-free structures were observed (Figure 12a,b). Despite the difference in the applied UAM parameters, the laminates had comparable shear strengths of ~41 MPa. Those laminates manufactured using modes 4, 7, and 9 were characterized by porous structures (Figure 12c–e). For mode 3, the characteristic defect was the deformation of CFs (Figure 12f), which could indicate partial damage to the prepregs.

**Figure 13:**
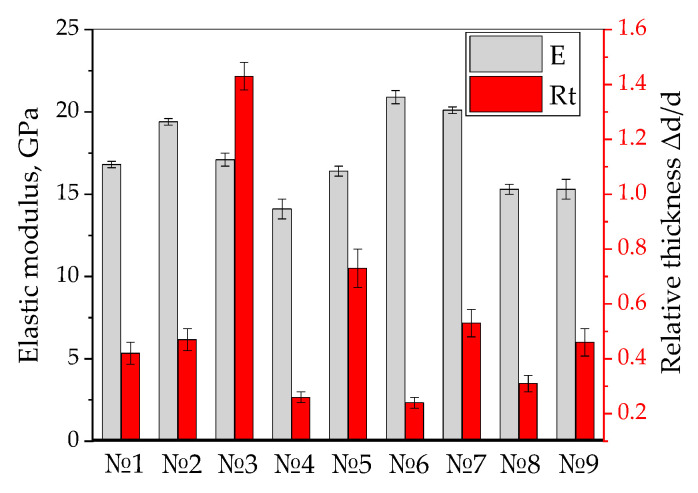
The elastic moduli and the relative thickness changes for the UAM laminates.

Figure 13 shows a bar diagram on the elastic moduli in the three-point bending tests and the relative thickness changes of the UAM laminates. It is evident that the applied UAM parameters did not lead to multiple changes in the elastic moduli. Although this characteristic was not taken into account in the calculations according to the Taguchi method, it was used for subsequent neural network modeling, since it characterized the stiffness of the laminates.

According to the authors, the extra resin layers were completely squeezed at *Rt* = 1, but only partly at *Rt* < 1; at *Rt* > 1, while the prepregs were additionally damaged in the UAM process. The ‘relative thickness change’ parameter was used for artificial neural network (ANN) modeling, since it allowed a holistic analysis of information about possible structural changes upon UAM of the laminates.

### 3.3. Artificial Neural Network (ANN) Modeling

The optimization of the UAM parameters using the obtained results could be considered as a sequential solution of two problems: (i) the approximation of the experimental data, and (ii) search for the region of suboptimal parameters in the space of approximated characteristics [25].

In the conducted experiments, the set of the UAM parameters was limited to the following: (i) the ‘extra resin layer thickness’, (ii) ‘UAM duration’, and (iii) ‘clamping pressure’ ones. An analysis of possible values was performed for each of them. As a result, their limits were identified (Table 5), within which further studies were performed.

To analyze the properties of the laminates, their mechanical (shear strength and elastic modulus) and structural (relative thickness change and porosity) characteristics (as output parameters) were selected. Both shear strength and elastic modulus were determined from the displacement–shear strength diagrams. The relative thickness changes were calculated according to Equation (1), by measuring the laminate thicknesses. Porosity was assessed visually from the photographs of their structures, and the following designation was adopted as a (semi)numerical characteristic: 0—‘joint not formed’; 1—‘incomplete joining’; 2—‘defect free structure; 3—‘presence of pores’; and 4—‘excessive deformation’. The limits of possible changes in both parameters and characteristics and the region of the optimal characteristics are presented in Table 5.

At the first stage, the experimental data obtained by the Taguchi method were analyzed (Table 3). To solve the approximation problem, the multiple linear regression method was initially applied. Based on this approach, linear dependencies of each characteristic on all UAM parameters were drawn. The most accurate regression model was obtained for the ‘shear strength’ factor, which possessed acceptable statistics: a determination coefficient R^2^ of 0.75 and a standard deviation of 2.17 MPa. The regression model for the ‘elastic modulus’ had the lowest statistics: a determination coefficient R^2^ of 0.02 and a standard error of 2.99 GPa. The latter completely rejected the possibility of using linear models to approximate the experimental data and proved the need to use nonlinear models. As shown in [27], the most effective method for approximating was artificial neural network modeling.

A model was based on a feedforward neural network (FFNN) with one hidden layer. A training sample was formed from the experimental data (9 parameter and 15 feature vectors) obtained by the Taguchi method (Table 3), with normalization to the space of acceptable limits (Table 5). Some models were synthesized with the enumeration of activation functions and the number of neurons in the hidden layer.

**Figure 14:**
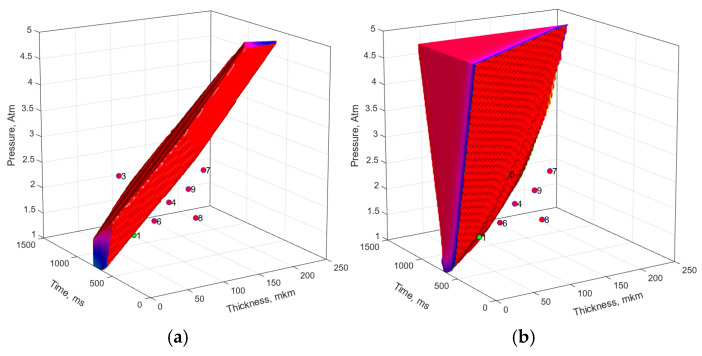
The experimental parameters and the suboptimal parameter regions drawn using the following: (**a**) the linear regression model (MSE = 0.01513, R^2^ = 0.2947), and (**b**) the FFNN model No. 1 (MSE = 0.0025068, R^2^ = 0.91035).

The fastest training with the greatest approximation accuracy characteristics was achieved when selecting the logarithmic tangent as the activation functions. The number of neurons was selected based on the results of an analysis of the complexity of the suboptimal parameter (SOP) region. Among all the models, the network including four neurons of the hidden layer with the simplest SOP region was selected (Figure 14b). For comparison, Figure 14a shows the SOP region plotted using the linear regression model (LRM). The FFNN model demonstrated the SOP region limited only on one side by a surface close to linear. Both models (regression and neural) were not limited in the parameter space and, therefore, were not physically justified. It should be noted that, despite significant shortcomings of these models, the FFNN was characterized by high statistical indicators: MSE = 0.0025068 and R^2^ = 0.91035. It should be noticed that due to the small sample size as well as the impossibility to form a testing sample, the estimates were obtained over the training sample data. These contradictions indicated the inappropriateness of these statistical estimates provided to demonstrate the accuracy of the neural network predictions and the need to develop new evaluation criteria.

Before the next modeling stage, it was decided that additional experimental studies are required with the use of a priori knowledge [28] at higher clamping pressure (Table 6).

**Figure 15:**
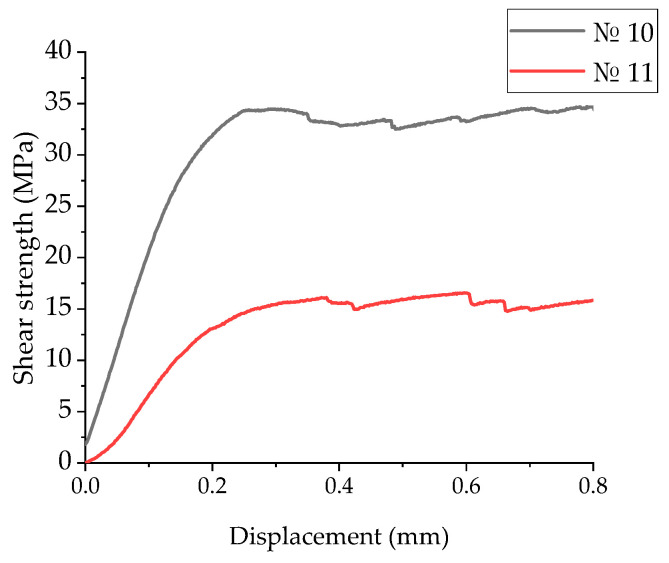
The displacement–shear strength diagrams for the UAM laminates fabricated using modes 10 and 11.

Figure 15 shows the “displacement–shear strength” diagrams of the laminates No. 10 and No. 11, recorded in the three-point bending tests. In these cases, pronounced irreversible (plastic) strains were observed. The maximum stresses were achieved at displacements of 0.25 mm for mode 10 and 0.38 mm for mode 11. Upon reaching the peak values, they practically did not decrease, reflecting the suppression of a main crack nucleation.

**Figure 16:**
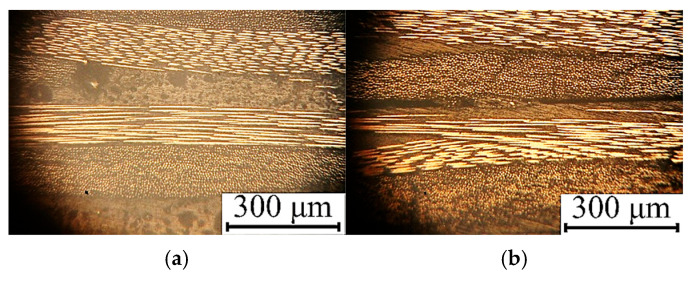
The optical photographs of the cross-sections of the UAM laminates fabricated using modes 10 (**a**) and 11 (**b**).

Table 7 presents the properties of the UAM laminates fabricated using modes 10 and 11, while Figure 16 shows photographs of their cross-sections. For mode 10, it possessed a high shear strength despite the great porosity (Figure 16a). In contrast, a significant decrease in the shear strength was observed for mode 11 due to some lack of fusion regions (Figure 16b). The data obtained in this additional experiment were also added to the training sample.

A priori knowledge was formulated according to the following assumptions:

1. The UAM process could not develop at short durations, as well as at insufficiently low or excessively high clamping pressures. In such cases, both shapes and dimensions of laminates would be unchanged, while their shear strengths would be minimal;
2. At too-long UAM durations, laminates would be partially or completely damaged.

**Figure 17:**
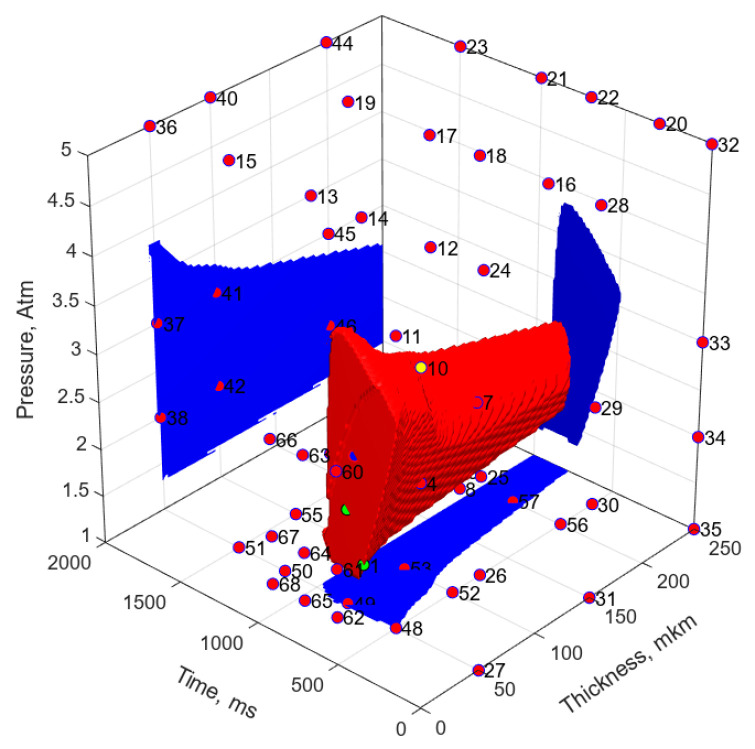
The experimental parameters and the suboptimal parameter regions drawn using the FFNN models 2. The number of neurons in the hidden layer was 6, and the training sample sizes were 21.

Based on the above a priori knowledge, new data were added to the training sample, the volume of which was 78 vectors. Some other FFNN models were synthesized, selecting both activation functions and the number of neurons that ensured the simplicity of the SOP region and acceptable learning accuracy. Figure 17 shows the SOP region plotted using the new FFNN 2 model. According to these data, the addition of a priori knowledge to the training sample led to a limitation of the SOP region in the planes where the vectors of a priori knowledge were defined. Thus, this model became closer to the physically justified SOP region.

Despite the fact that the SOP region exhibited an extended pattern, prolonging beyond the analysis area of the ‘extra resin layer thickness‘ parameter, high strength characteristics and the required structural properties were predicted by the model in its “low-level” (~50 μm) region. For this reason, a decision was made to conduct verification experiments at the next stage, using the UAM parameters presented in Table 8.

**Figure 18:**
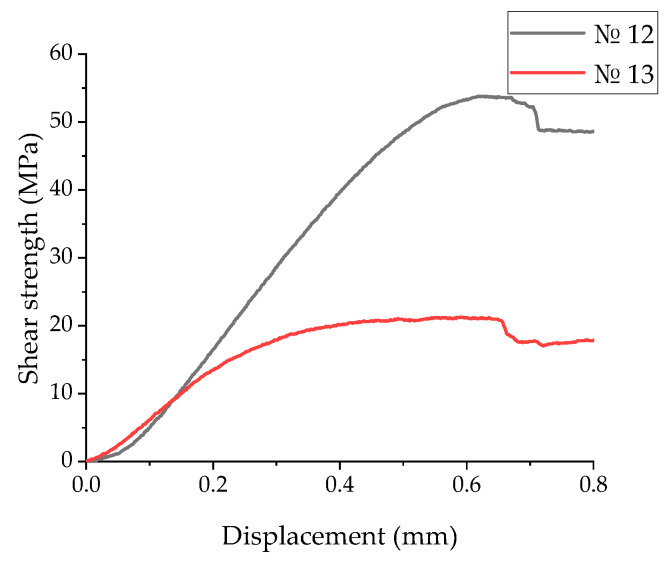
The displacement–shear strength diagrams for the UAM laminates fabricated using modes 12 and 13.

Figure 18 shows the displacement–shear strength diagrams, which differed significantly. The maximum stresses were achieved at displacements of 0.65 mm for mode 12 and 0.61 mm for mode 13.

**Figure 19:**
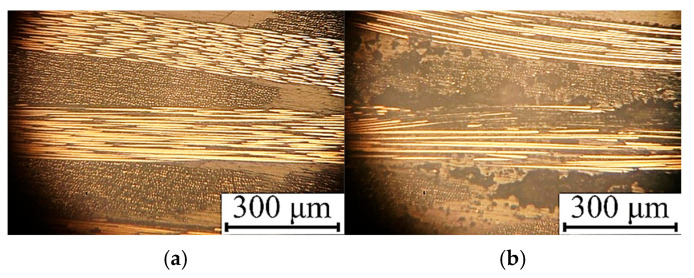
The photographs of the cross-sections of the UAM laminates fabricated using modes 12 (**a**) and 13 (**b**).

The results of the experimental evaluation of the output parameters of the UAM laminates are presented in Table 9, while Figure 19 shows photographs of their cross-sections. Mode 12 ensured the achievement of the maximum both shear strength and (relative) thickness change, since the laminate structure was homogeneous and visually defect-free (Figure 19a). For mode 13, a low shear strength was recorded because of the damaged prepregs, while discontinuities were observed at the interlaminar interfaces due to the high UAM duration (Figure 19b).

The models were verified using the experimental results. The predicted laminate characteristics are given in Table 10. The mean of normalized deviations (MNDs) of the predicted values from the experimental data were calculated as estimates of the forecast accuracy. For all models, the MND levels varied widely. Due to the small amount of data, it was not possible to provide statistical estimates or draw general conclusions based on these results. However, the authors considered this task as a classification of the characteristics of the UAM laminates into the optimal/other types (according to the ranges in Table 5). Respectively, the reliability of the models was determined by a larger amount of data and could be calculated as follows:

(2) $$p=\frac{1}{NM}\sum_{n=1}^{N}\sum_{m=1}^{M}\left\lceil Y_{m}^{d}\leq y_{n,m}\leq Y_{m}^{u}\right\rceil ,$$

where $y_{n,m}$ was the *n-*th predicted value of the *m*-th characteristic for the *n*-th combination of the parameters; $\left[Y_{m}^{d},Y_{m}^{u}\right]$ was the range of the optimal values of the *m*-th characteristic; and $\left\lceil \ldots \right\rceil$ was the transformation of both logical ones (true) and zeros (false) into numbers. So, a comparison of the simplest LRM and FFNN 1 models, based on the results obtained by the Taguchi method, and the FFNN 2 one, trained using the extended sample with a priori knowledge, indicated a clear advantage of the latter.

**Figure 20:**
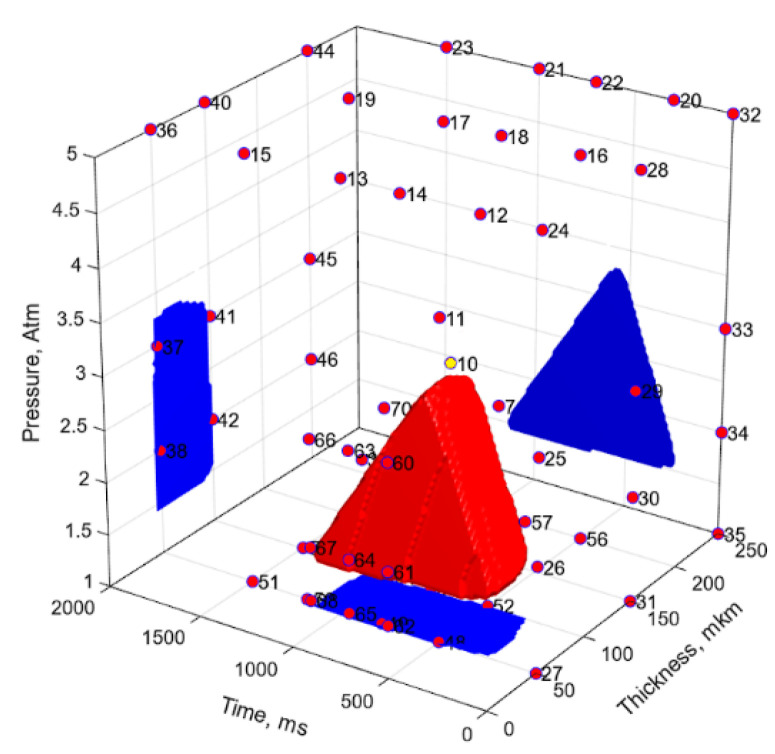
The experimental parameters and the suboptimal parameter regions drawn using the FFNN models 3. The number of neurons in the hidden layer was 6, and the training sample sizes were 23 experimental vectors + 57 a priori knowledge ones.

At the final stage, the FFNN 2 model was additionally trained using the experimental results obtained according to modes 12 and 13, and the SOP region was justified (Figure 20). The following combination of the UAM parameters was used as the optimal model parameters: an extra resin layer thickness of 47.7 μm, an UAM duration of 752 ms, a clamping pressure of 2.61 atm, as well as the predicted laminate properties: a shear strength of 52.2 MPa, a relative thickness change of 0.99988, a porosity of 2.8, and an elastic modulus of 21.5 GPa. The predicted parameters almost coincided with those obtained for mode 12 (Table 8 and Table 9) with the prognosis accuracy of MND = 0.041.

**Figure 21:**
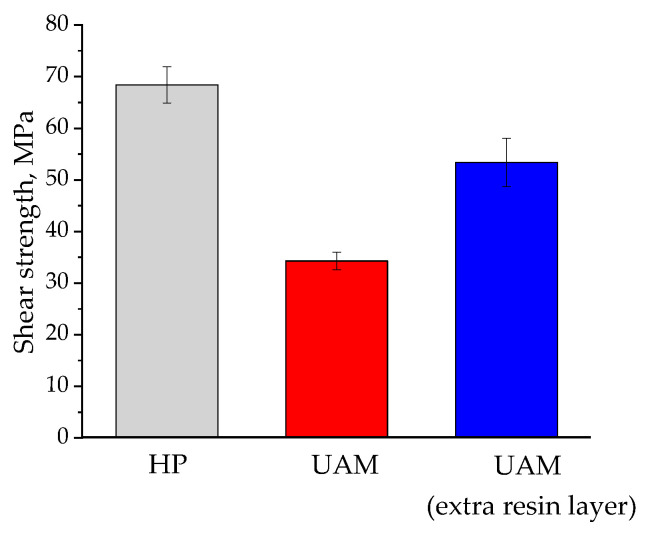
The shear strength values for the HP and UAM laminates.

Figure 21 shows the bar diagram on shear strength values, enabling us to conclude that the UAM method can be used to produce laminates with improved mechanical characteristics (within 20%) being comparable with those of the TF ones.

**Figure 22:**
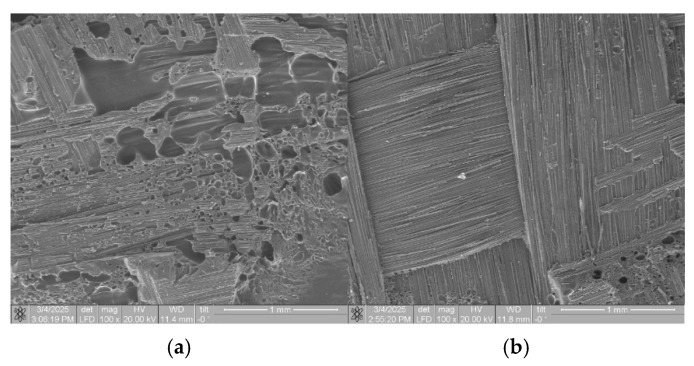
The SEM micrographs of the structures at the interfaces between layers (prepregs) of the UAM laminates (PEEK/CF70 %) without (**a**) and with the extra resin layer No. 12 (**b**).

Figure 22 shows SEM micrographs of the interlaminar interface (between the repregs) of the UAM laminates without and with the extra resin layer. It was evident that the applied approach allowed for minimizing the number of defects at the interfaces region.

Table 11 presents the results of the evaluation of the shear strengths of some laminates manufactured by different methods. In this scientific area, most studies were devoted to the CF/PEEK ones. According to these data, the UAM method enabled us to fabricate the CF/PEEK (PEI) laminates possessing properties not inferior to those reported earlier.

## 4. Discussion

**Figure 23:**
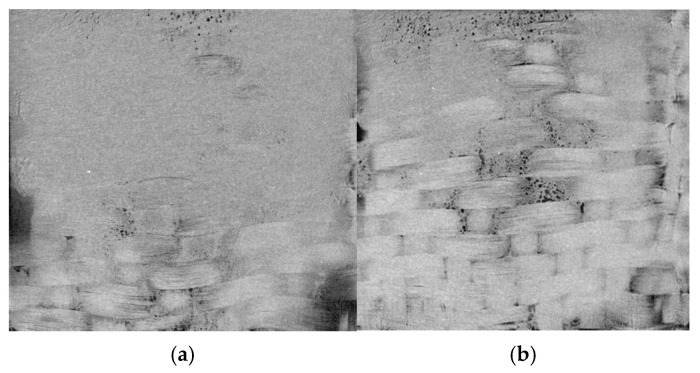
The CT examination of the interlaminar border in US-consolidated laminate; fiber weight fraction 55 wt.% (**a**) and 70 wt.% (**b**).

It should be noticed as known from the literature and studies by the authors that US welding does not ensure the complete and uniform bonding of the adherends at the interlaminar interface [35,36,37,38]. This is related to the fact that it is impossible to evenly focus US-energy over the square area of 20 × 20 mm^2^ (Figure 23). This was also shown by the data of computational tomography in our previous study on the topic [39]. In addition, the lack of polymer binder in the prepreg hindered the appropriate consolidation of the adjacent layers. This phenomenon was examined numerically in a paper by the authors [40], where the effect of bonding area on the flexural modulus and strength was estimated with the use of FEM.

Since the UAM is almost controlled by friction heating, the local temperature and bonding pressure affect the consolidation process. In this concern, the lower the clamping pressure, the faster the melting of the polymer binder develops. However, the heating proceeds in nonlinear way, which was numerously shown in the literature [38,41,42]. In doing so, the temperature in the US-consolidation zone can reach 280–350 °C.

Two main competing processes affect structure formation during the US-consolidation: The increase in polymer weight fraction improves interlaminar adhesion due to the even distribution of the binder as well as the wetting of the contacting prepregs. On the other hand, the decrease in the fiber volume fraction gives rise to diminishing the strength of the composite. For this particular reason, by increasing the weight fraction of the polymer at the interlaminar interface, the strength properties of UAM composites were enlarged.

The mechanism that led to the brittle failure observed at 70 wt.% CF in TF laminates is related to both stress concentration and reduced matrix content. First, because of the high level of external applied stress (ISS = 70 MPa), a stress concentrator occurs to be relaxed with main crack propagation. Then, the lack of polymer binder does not ensure due level of cracking resistance.

In contrast to the TF method, the UAM laminate with 70 wt.% CF exhibited a low strength and pronounced ductility. The reason was the deficiency of polymer binder preventing the formation of reliable interlaminar bonding. It is the lack of fusion that promotes the development of inelastic deformation. The TF laminate with the equal weight fraction of the fibers deformed elastically with a brittle pattern of failure at reaching the ultimate strength (a certain arrest of the main crack happened when it reached the interlaminar interfaces).

Table 12 illustrates data on fracture energy measured under three-point bending tests. It was calculated through the area beneath the corresponding loading diagram. It has been shown that the highest value of fracture energy is appropriate for the TF laminate with 65 wt.%. CF. The largest value of this parameter was appropriate for the UAM laminate enforced with 60 wt.% CF. Thus, this parameter might be used for the selection of the best weight fraction of the CF in the UAM laminate.

The nonlinear behavior of the curve in Figure 10 is determined by a couple of factors. Firstly, the nonlinear nature of polymer binder heating up at UAM. For doing so, various contents of the polymer in a prepreg gave rise to different melting degrees under identical conditions of applying US-vibrations. Secondly, being directly connected to the first factor, various patterns of melted binder flowing due to its different weight fraction and degree of melting.

In this study, the presence of pores was interpreted as the incorrect selection of process parameters. In addition, the presence of pores testifies for the nonevent interlaminar interface structure. The problem of the relation between the ISS and porosity is of actuality. However, it was not studied in detail in this paper.

The UAM is surely a more damaging method in contrast to TF due to the (i) employed way of frictional heating and (ii) higher clamping pressure, as well as the (iii) fast process development. For this reason, the UAM under mode 3 (Figure 12f) must be accompanied with thermal or mechanical damaging. This is the reason for the lower ISS at UAM. However, the selected optimal process parameters of the UAM makes it possible to minimize the level of such damage.

## 5. Conclusions

In this study, both TF and UAM laminates with CF contents from 55 to 70 wt.% were fabricated and investigated. Based on the obtained results, the following conclusions were drawn:

1. The UAM laminates with CF contents above 55 wt.% possessed shear strengths lower by 40% in comparison with those of the TF ones, due to the insufficient amount of the binder in the prepregs to form reliable interlaminar joints;
2. For enhancing the shear strength of UAM laminates up to the levels of the TF ones, extra resin layers with thicknesses of 50, 100, and 150 μm were deposited. By ranking the UAM parameters using the Taguchi L9 method, it was possible to increase the shear strengths by 30% relative to those of the trial laminates. Further improvements were achieved by artificial neural network modeling;
3. According to the results obtained by the artificial neural network modeling, the use of the 50 µm thick extra resin layer allowed us to increase the shear strengths up to 50% relative to those of the trial laminates at a CF content of 70 wt.%. This improvement was achieved via minimizing the number of defects at the interlaminar interfaces;
4. The dependences of both mechanical and structural characteristics of the laminates on the UAM parameters were essentially nonlinear. For their analysis and the optimization of the UAM parameters, direct propagation neural networks with the minimal architecture were used. Under the ultra-small sample conditions, the use of a priori knowledge enabled us to predict the results rather accurately.

## Figures and Tables

**Table 1:** The CF contents and the thicknesses of the prepregs.

Fiber Weight Fraction, %	Fiber Volume Fraction, %	Prepreg Thickness, µm
70 ± 2	63 ± 2	300 ± 25
65 ± 2	58 ± 2	350 ± 25
60 ± 2	53 ± 2	400 ± 25
55 ± 2	48 ± 2	450 ± 25

**Table 2:** The level numbers and the values of the Taguchi factors according to the L9 format.

Experiment Number	Levels and Values of (Technological) Factors		
	Extra Resin Layer Thickness *d*, μm	USW Duration *t*, ms	Clamping Pressure *P*, atm
1	50	700	1.5
2	50	800	2.0
3	50	900	2.5
4	100	700	2.0
5	100	800	2.5
6	100	900	1.5
7	150	700	2.5
8	150	800	1.5
9	150	900	2.0

**Table 3:** The properties of the UAM laminates.

No.	Shear Strength, MPa	Thickness Change *Δd*, µm
1	40.6 ± 0.7	125 ± 15
2	37.0 ± 0.6	140 ± 15
3	33.8 ± 0.4	430 ± 30
4	43.5 ± 1.2	155 ± 30
5	41.3 ± 0.6	440 ± 20
6	38.1 ± 1.4	145 ± 15
7	45.2 ± 1.9	480 ± 10
8	39.1 ± 0.8	280 ± 30
9	43.2 ± 0.7	415 ± 15

**Table 4:** The results of ranking both influence factors and levels for the UAM laminates.

Level	Shear Strength, MPa			Thickness Change Δ*d*, µm		
	*d*	*t*	*p*	*d*	*t*	*p*
1	31.38	36.69	31.88	45.91	46.55	44.76
2	32.24	31.84	32.28	46.67	48.24	46.39
3	32.55	31.64	32.00	51.64	49.42	53.05
4	1.17	1.05	0.41	5.74	2.87	8.29
5	1	2	3	2	3	1

**Table 5:** Areas of the analysis of the UAM parameters, the laminate characteristics, and the optimal UAM parameters.

	Acceptable Limits		Optimal Range	
	Min	Max	Min	Max
Parameters				
Extra resin layer thickness *d*, μm	0	250		
UAM duration *t*, ms	0	1500		
Clamping pressure *P*, atm	1	5		
Characteristics				
Shear strength, MPa	0	60	35	60
Relative thickness change *Rt*	0	2	0.82	1.05
Porosity	0	4	2	3.1
Elastic modulus, GPa	0	30	15	30

**Table 6:** The additional experiment parameters.

Experiment Number	Extra Resin Layer Thickness *d*, μm	UAM Duration *t*, ms	Clamping Pressure *P*, atm
10	100	700	3.2
11	50	500	4.0

**Table 7:** The output parameters for the UAM laminates.

Experiment Number	Shear Strength, MPa	Thickness Change Δ*d*, µm	Elastic Modulus, GPa	Relative Thickness Change, *Rt*
10	34.9 ± 3.1	330 ± 20	20.5 ± 0.8	0.55
11	14.5 ± 4.2	120 ± 20	6.1 ± 2.7	0.40

**Table 8:** The verification experiment parameters.

Experiment Number	Extra Resin Layer Thickness *d*, μm	UAM Duration *t*, ms	Clamping Pressure *P*, atm
12	50	750	2.6
13	100	1050	2.6

**Table 9:** The output parameters for the UAM laminates No. 12 and 13.

Experiment Number	Shear Strength, MPa	Thickness Change Δ*d*, µm	Elastic Modulus, GPa	Relative Thickness *Rt*
12	53.4 ± 4.7	200 ± 30	20.0 ± 0.7	0.91
13	20.5 ± 0.5	360 ± 30	15.1 ± 1.1	0.60

**Table 10:** The predicted values of the models.

Experiment Number	Model	Shear Strength, MPa	Relative Thickness Change	Porosity	Elastic Modulus, GPa	MND	Classification Reliability
10	LRM	42.7882	0.993	2.1727	16.8655	0.090	0.562
11		44.5494	1.1534	2.5687	16.2040	0.343	
12		38.6165	1.0324	2.3783	17.1999	0.139	
13		34.4574	1.0018	1.6864	18.1827	0.244	
10	FFNN 1	41.2134	0.8876	2.8687	13.6558	0.140	0.562
11		40.2628	0.922	2.9703	15.2465	0.313	
12		38.5479	0.9827	2.9983	20.7444	0.077	
13		35.2201	1.0336	2.0002	17.4245	0.225	
12	FFNN 2	39.919	0.978	2.715	21.240	0.093	0.875
13		9.820	0.803	0.023	0.000	0.428	

**Table 11:** The shear strength values for both CF/PEI and CF/PEEK laminates.

Manufacturing Method	Materials	Shear Strength, MPa	Relative Thickness
Thermoforming	CF/PEI unidirectional prepreg tape; matrix content is 37 wt.%	86.7	[29]
Hot air automated fiber placement	CF/PEI unidirectional prepreg tape; matrix content is 42 wt.%	28.0	[30]
Hot gas torch automated fiber placement	CF/PEEK unidirectional prepreg tape, fiber volume fraction of 60%, and thickness of 0.163 mm	23.0	[31]
Laser automated fiber placement	CF/PEEK unidirectional prepreg tape, fiber volume fraction of 60%, and thickness of 0.150 mm	33.0	[32]
Laser automated fiber placement	CF/PEEK unidirectional prepreg tape; thickness of 0.140 mm	58.6	[33]
Laser automated fiber placement	CF/PEEK unidirectional prepreg tape; resin weight fraction is 34%	70.3	[34]

**Table 12:** Data on of fracture energy measure under 3-point bending tests.

Composite Type	Fracture Energy, MJ/m^3^
TF—70 wt.%	18.8 ± 0.7
TF—65 wt.%	33.2 ± 1.2
TF—55 wt.%	17.1 ± 1.0
UAM—70 wt.%	15.7 ± 0.7
UAM—65 wt.%	15.1 ± 0.5
UAM—60 wt.%	20.0 ± 0.5
UAM—65 wt.%	16.0 ± 0.6

## Data Availability

The data presented in this study are available on request from the corresponding author. The data are not publicly available due to confidential disclosure reasons.
